# A Review of Classification, Biosynthesis, Biological Activities and Potential Applications of Flavonoids

**DOI:** 10.3390/molecules28134982

**Published:** 2023-06-25

**Authors:** Shen Chen, Xiaojing Wang, Yu Cheng, Hongsheng Gao, Xuehao Chen

**Affiliations:** 1 School of Horticulture and Landscape Architecture, Yangzhou University, Yangzhou 225009, China; chenshen999@126.com (S.C.);; 2 Key Laboratory of Plant Resource Conservation and Germplasm Innovation in Mountainous Region (Ministry of Education), Institute of Agro-Bioengineering, Guizhou University, Guiyang 550025, China

**Keywords:** flavonoids, biosynthesis pathway, classification, biological activity, application

## Abstract

Flavonoids represent the main class of plant secondary metabolites and occur in the tissues and organs of various plant species. In plants, flavonoids are involved in many biological processes and in response to various environmental stresses. The consumption of flavonoids has been known to reduce the risk of many chronic diseases due to their antioxidant and free radical scavenging properties. In the present review, we summarize the classification, distribution, biosynthesis pathways, and regulatory mechanisms of flavonoids. Moreover, we investigated their biological activities and discuss their applications in food processing and cosmetics, as well as their pharmaceutical and medical uses. Current trends in flavonoid research are also briefly described, including the mining of new functional genes and metabolites through omics research and the engineering of flavonoids using nanotechnology. This review provides a reference for basic and applied research on flavonoid compounds.

## 1. Introduction

Flavonoids, a group of important secondary metabolites, are widely distributed in widely found in fruits, vegetables, herbs, stems, cereals, nuts, flowers, and seeds [1]. So far, over 10,000 flavonoid compounds have been isolated and identified [2]. Flavonoids processed favorable biochemical and antioxidant effects on various diseases such as cardiovascular disease, cancer, and neurodegenerative disease [3]. Flavonoids are associated with a broad spectrum of health-promoting effects and are an indispensable component in a variety of nutraceutical, pharmaceutical, medicinal and cosmetic applications. This is mainly attributed to their anti-inflammatory, anti-oxidative, anti-carcinogenic, and anti-mutagenic properties as well as the ability to regulate key cellular enzyme function [4,5].

Flavonoids are synthesized through the phenylpropanoid metabolic pathway and possess 15 carbon atoms arranged in three rings (C6–C3–C6) labeled A, B, and C [6]. Flavonoids play a variety of biological activities in plants, animals, and bacteria [7]. In plants, flavonoid compounds are normally found in flowers, fruits, leaves, and seeds, which are responsible for their color, fragrance, and flavor characteristics [8]. As important secondary metabolites, plant flavonoids are involved in regulating auxin transport, male fertility, pollination, seed development, flower coloring, and allelopathy [9]. Flavonoids play protective roles against abiotic (ultraviolet radiation, cold, salt, drought, and heavy metals) and biotic (herbivores, bacteria, fungi) stresses [10]. Flavonoids are classified into various types depending on their chemical structure, degree of unsaturation, and oxidation of carbon ring, including flavones, flavanones, isoflavones, flavonols, chalcones, flavanols, and anthocyanins. Each of these flavonoids is widely distributed in nature [4,11].

Flavonoids have attracted increasing research interest in recent years. Thus, in the present review, we systematically summarize recent progress in plant flavonoid research in terms of their classification, biosynthesis, biological activity, and potential applications in food processing, cosmetics, and pharmaceutical industries [5,12,13]. We also discuss the use of omics research for mining functional genes and identifying new flavonoid compounds. In addition, we summarize recent research progress on the role of flavonoid nanoparticles in various diseases. The main purpose of this review is to provide up-to-date information on the classification, biosynthesis, biological activity, and potential applications of flavonoids. In addition, research into the therapeutic potential of flavonoid nanoparticles has highlighted their value and importance in preventing life-threatening diseases and improving global health.

## 2. Methodology

**Figure A1:**
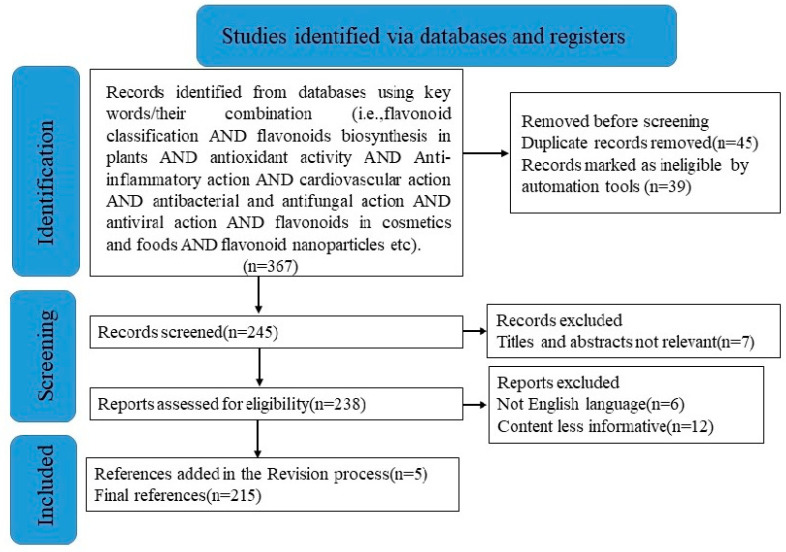
PRISMA flow chart presenting literature data selection.

To perform a comprehensive review of plant flavonoids, emphasizing the classification, biosynthesis and regulatory mechanisms, biological activities, potential applications, omics research, and flavonoid nanoparticles, a thorough literature search was performed. Seven scientific electronic databases, including SCiFinder, PubMed, MEDLINE, EMBASE, Scopus, ScienceDirect, and the Google Scholar library, were used for searching in PubMed. The keywords were searched alone or in combination with other keywords. The PRISMA flow chart was used to display the methodology for the published information selection through Page’s method (Figure A1) [14,15].

## 3. Flavonoid Classification

**Figure 1:**
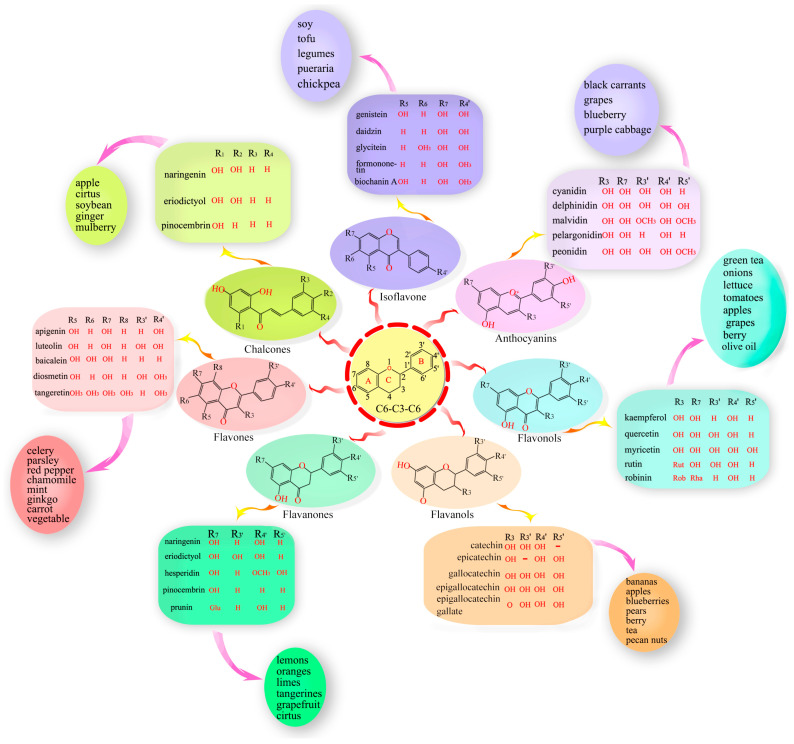
The basic molecular structure of flavonoids, classification, and distribution in various plants.

Flavonoids are mainly found in plant cell vacuoles in the form of *C*-glycosides or *O*-glycosides [16]. The basic molecular structure of flavonoids depends upon their basic C6–C3–C6 skeleton (labeled A, B, and C, Figure 1). Flavonoids are classified into seven subclasses based on modifications to their basic skeletons; these subclasses include flavones, flavanones, isoflavones, flavonols, chalcones, flavanols, and anthocyanins [17,18].

### 3.1. Flavones

**Figure A2:**
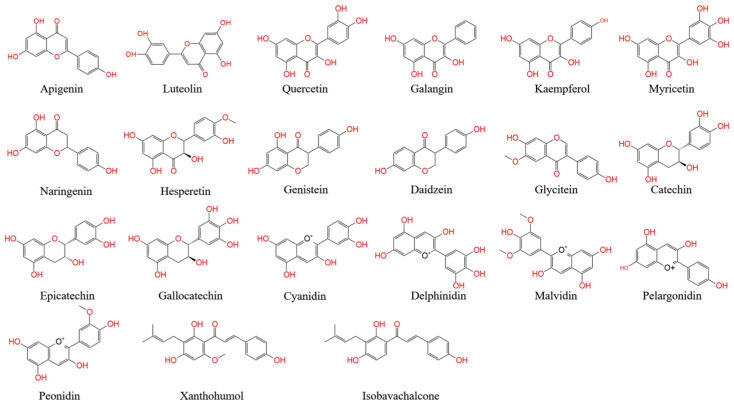
The Basic Structure of Flavonoids.

Flavones, one of the largest classes of flavonoids, consist of 4H-chromen-4-one bearing a phenyl substituent at position 2. Flavones mostly occur as 7-*O*-glycosides, which are found in celery, parsley, red pepper, chamomile, mint, and ginkgo [5,19,20]. Apigenin and luteolin are two common flavones (Figure A2). In nature, apigenin is usually found in a glycosylated form, with a sugar moiety attached to the tricyclic core structure via hydroxyl groups (*O*-glycosides) or directly to carbon (*C*-glycosides) [21]. The principal ingredients of apigenin are glycosylated apiin, apigenin, vitexin, isovitexin, or rhoifolin. Apigenin can scavenge free radicals and regulate antioxidant enzyme activity in pancreatic cells, and apigenin can decrease inflammation in cancer, neuroinflammation, and cardiovascular diseases [9,22].

### 3.2. Flavonols

Flavonols, also called 3-hydroxy flavone, can be identified by specific substitutions in their A- and B-rings, which are connected by a three-carbon chain [23]. Flavonols possess hydroxyl groups at positions 5 and 7 in the A-ring and are mainly present in epidermal cells to protect DNA against UV-induced damage [24]. Four types of flavonol compounds (quercetin, galangin, kaempferol, and myricetin) are mainly distributed in vegetables and fruits, such as asparagus, onions, lettuce, broccoli, tomato, and apples (Figure A2) [25]. Flavonols exhibit interesting biological activities, including antioxidant, antibacterial, cardioprotective, anticancer, and antiviral activities. Dietary flavonols can significantly decrease the risk of gastric cancer in smokers and in women (Figure A2).

### 3.3. Flavanones

Flavanones (dihydro-flavones) possess a saturated C-ring [26]. The saturated double bond between positions 2 and 3 in the C-ring represents the only structural difference between flavanones and other flavonoid compounds [27]. Flavanones are mainly distributed in citrus fruits, including oranges, lemons, mandarins, grapefruits, clementines, and limes [28]. Flavanones contain hydroxyl groups at positions 5 and 7 in the A-ring and possess hydroxyl/methoxy substituents at the C3 or C4 positions of the B-ring [29]. The defining characteristic of flavanones is a disaccharidic moiety linked to the seven positions of aglycone [30]. Depending on their structural differences, flavanones can occur in the form of naringin, naringenin, hesperidin, hesperetin, pinocembrin, likvirtin, and eriodictyol [31]. Among them, naringenin and hesperetin, as the main dietary flavanones, occur almost exclusively in citrus fruits (Figure A2) [28,32]. Naringin can increase the activity of antioxidant enzymes (CAT, PON, GPx, and SOD) and enhance the immune system. Furthermore, naringenin and hesperetin have been shown to recover impaired thyroid function in rats.

### 3.4. Isoflavonoids

Isoflavones have a B-ring at the C3 position of the heterocyclic C-ring of the diphenylpropane (C6–C3–C6) backbone, which represents their only chemical structural difference from other flavonoids [33]. Isoflavonoids are characteristic metabolites of leguminous plants and play essential roles in nodule induction and microbial signaling in legumes [34,35]. Isoflavones are classified into three groups: genistein, daidzein, and glycitein (Figure A2) [36]. The molecular structure of isoflavones is similar to that of animal estrogens. Isoflavones are phytoestrogens that exhibit potent estrogenic activity. Phytoestrogens are similar in structure to the human female hormone 17-β-estradiol in that they bind to estrogen receptors [37]. In addition, isoflavones possess a strong antioxidant activity, which can decrease the risk of cancers by inhibiting free radical-induced DNA damage [37].

### 3.5. Flavanols

Flavanols, also called catechins or flavan-3-ols, are characterized by a hydroxyl group at position 3 in the C-ring [38]. Flavanols lack a double bond between positions 2 and 3 in the C-ring [39]. Several flavanols, including catechin, gallocatechin 3-gallate, gallocatechin, epicatechin, epicatechin 3-gallate, catechin 3-gallate, and epicatechin 3-gallate, are widely distributed in many fruits (e.g., apples, bananas, pears, and blueberries) [40,41]. Flavanols can protect blood vessels against tobacco by increasing the content of NO in blood vessels. A flavanol-rich diet can facilitate the permanent improvement of endothelial function and prevent the development of cardiovascular diseases [42,43].

### 3.6. Anthocyanins

Anthocyanins, as glycosylated polyphenolic compounds, are a group of soluble vacuolar pigments that possess a range of colors, from orange, red, and purple to blue, depending on the pH of the micro-environment of the flowers, seeds, fruits, and vegetative tissues [44]. The position and number of hydroxyl and methoxyl groups present as substituents in the flavylium structure result in different anthocyanins (Figure A2). Thus, over 650 anthocyanins have been identified in many plants [45]; these are grouped into five items, including cyanidin, delphinidin, malvidin, pelargonidin, and peonidin, and their corresponding derivatives [46]. Anthocyanins are mainly found in the outer cell layer of various fruits and vegetables, such as blackcurrants, grapes, and berries [47,48]. The antioxidant ability of anthocyanins is associated with their ring orientation and the position and number of free hydroxyls around the pyrone ring. Anthocyanins play important roles in visual acuity, cholesterol decomposition, and the reduced risk of cardiovascular disease in humans [49,50]. In addition, anthocyanins are commonly used as food colorants.

### 3.7. Chalcones

Chalcones (1,3-diaryl-2-propen-1-ones) are natural open-chain flavonoids, carrying up to three modified or unmodified C5-, C10-, and C15-prenyl moieties on both their A and B-rings. These bioactive products are widely distributed in the Fabaceae, Moraceae, Zingiberaceae, and Cannabaceae families [9]. They exhibit a wide spectrum of pharmacological effects, including antioxidant, antibacterial, anthelmintic, antiulcer, antiviral, antiprotozoal, and anticancer effects [51]. Chalcones are precursors of flavonoids and isoflavonoids. Their structural features are easily constructed from simple aromatic compounds. Their prominent bioactivity has inspired the synthesis of chalcone analogs, as well as minor structural modifications to natural chalcones; these compounds form a large collection of bioactive chalcone derivatives [52]. Xanthohumol and isbavirachalone are two representative derivatives that exhibit abundant biological and pharmacological activity (Figure A2) [49].

Generally, the number and position of –OH groups have a great influence on flavonoid bioactivity. The –OH groups can link to the carbon atoms of the benzene ring (3,5,7, and 3′,4′-dihydroxy substitution pattern), which directly determines the bioactivity of flavonoids. Moreover, the position of the –OH group also influenced the flavonoid bioactivity. The most effective radical scavengers are flavonoids with the 3′,4′-dihydroxy substitution pattern on the B-ring and/or hydroxyl group at the C-3 position. In addition, the C2–C3 double bond is not necessary for high activity. Flavanols lacking the C2–C3 double bond displayed strong activity. The presence of a 3 –OH group significantly enhances the bioactivity.

## 4. Flavonoid Biosynthesis in Plants

### 4.1. Flavonoid Biosynthetic Pathways

**Figure 2:**
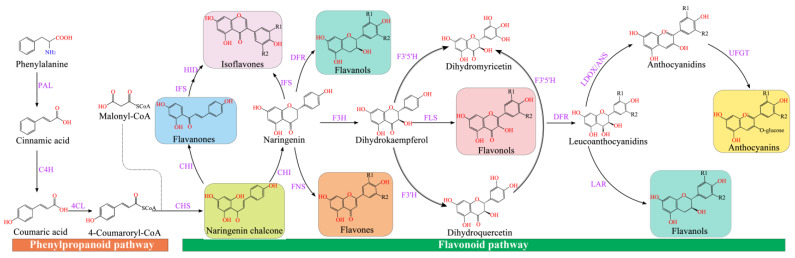
Flavonoid synthesis pathway. CHS (chalcone synthase) can catalyze three molecules of malonyl-CoA and one molecule of *p*-coumaroyl-CoA to form naringeninchalcone [56]. Malonyl-CoA is an important precursor for the synthesis of natural products, including flavonoids and polyketides [57]. CHI (chalcone isomerase) converted naringenin-chalcone into flavanones [58]. Naringenin, as an important flavonoid skeleton, is catalyzed by FNSI and FNS II (flavone synthase I and flavone synthase II) and IFS (isoflavone synthase) to form flavones and isoflavones, respectively [59]. Furthermore, flavanone-3-hydroxylase (F3H), flavonol 3′-hydroxylase (F3′H), and flavonol 3′5′-hydroxylase (F3′5′H) catalyzed naringenin to generate dihydro-myricetin, dihydro-kaempferol, and dihydro-quercetin, respectively [60]. The FLS (flavonol synthase) converted dihydroflavonols into flavonols (kaempferol, quercetin, and myricetin), which was catalyzed by the dihydroflavonol 4-reductase (DFR) to generate leucoanthocyanidins [61], which was catalyzed by leucoanthocyanidin dioxygenase (LDOX) to produce anthocyanidins [62]. Anthocyanidins and leucoanthocyanidins were further converted to proanthocyanidins catalyzed by leucoanthocyanidin reductase (LAR) and anthocyanidin reductase (ANR), respectively [63]. Modification of anthocyanins is responsible for the stabilization of vacuolar anthocyanins, including glycosylation, methylation, and acylation [64].

Flavonoid synthesis occurs at the junction of the shikimate pathway and the acetate pathway. The former can generate *p*-coumaroyl-CoA, and the latter regulates C_2_-chain elongation [53] (Figure 2). Phenylalanine ammonia-lyase (PAL) deaminates phenylalanine to ammonia and cinnamic acid [54]. Then, C4H (cinnamic acid 4-hydroxylase) catalyzes the production of 4-coumaric acid [4], and 4CL (4-coumaric acid: CoA ligase) converts 4-coumaric acid to form 4-coumaroyl-CoA, which is a key enzyme in the phenylpropanoid metabolic pathway that regulates the biosynthesis of lignin and flavonoids [55].

### 4.2. Transcriptional Regulation of Flavonoid Synthesis

Flavonoid biosynthesis is tightly regulated by biosynthetic enzymes and regulatory transcription factors (TFs) [65]. Several TF families have been reported to be involved in regulating flavonoid biosynthesis in plants, including WRKY, Dof, MADS-box, bZIP, MYB, bHLH, WD40, and NAC (Table 1) [66]. Plant MYBs are characterized by a highly conserved MYB DNA-binding domain and are further classified into four groups based on the position and number of MYB repeats: 1R-MYB, 2R-MYB, 3R-MYB, and 4R-MYB [67]. Among them, R2R3-MYB TFs are involved in regulating the expression of structural genes in the flavonoid pathway [68]. For example, transgenic tobacco overexpressing *NtMYB3* from *Narcissus tazetta* can reduce the content of flavonoids by inhibiting the expression of *FLSs* [69]. Transgenic *Arabidopsis* overexpressing *GbMYB2* from *Ginkgo biloba* can decrease flavonoid accumulation by inhibiting the expression of some structural genes (e.g., *GbPAL*, *GbFLS*, *GbANS*, and *GbCHI*) [70]. Yan et al. revealed that soybean *GmMYB100* negatively regulated flavonoid biosynthesis by inhibiting the activities of CHS and CHI promoters [71]. In addition, the overexpression of *PpMYB17* in pear calli was found to bind and activate the promoters of structural genes of *PpCHS*, *PpCHI*, *PpF3H*, *PpFLS*, and *PpUFGT* under light conditions, which enhanced the biosynthesis of flavonoids [72]. Transgenic tobacco overexpressing *FtMYB31* from *Fagopyrum tataricum* increased the expression of *CHS*, *F3H*, and *FLS* genes and promoted the accumulation of flavonoids [73]. The overexpression of *SbMYB8* from *Scutellaria baicalensis* in transgenic tobacco promoted the expression of the *SbCHS* gene, increased flavonoid content, and enhanced the activities of antioxidant enzymes in transgenic tobacco [63]. Furthermore, bHLH TFs play essential roles in regulating the biosynthesis of flavonoids. *CsMYC2* was able to promote flavonoid biosynthesis by increasing the expression of the *UFGT* gene [74]. *MdbHLH3* promoted anthocyanin accumulation and fruit coloration in response to low temperatures in apples [75]. In addition, MBW complexes (MYB-bHLH-WD40) regulate flavonoid biosynthesis in different plants [64,76]. The TT2–TT8–TTG1 complex plays a major role in developing seeds and also plays an important role in regulating the expression of LBGs (*DFR*, *LDOX*, *TT19*, *TT12*, *AHA10*, and *BAN*) [77]. Moreover, the MBW complex exhibits tissue-specific regulation of the expression of the genes involved in flavonoid biosynthesis [78]. The MYB5–TT8–TTG1 complex is active in the endothelium, regulating *DFR*, *LDOX*, and *TT12* expression, whereas the TT2–EGL3/GL3–TTG1 complexes regulate the expression of *LDOX*, *BAN*, *AHA10*, and *DFR* in the chalaza [78].

In addition, several TF families, including bZIP, NAC, Dof, and WRKY, play important roles in regulating flavonoid biosynthesis [79,80]. For example, *VvibZIPC22* was able to bind and activate the promoters of structural genes of *VviCHI* and *VviCHS* to increase their flavonoid contents [81]. Transgenic tobacco overexpressing *NtHY5* increased the expression of phenylpropanoid pathway genes, promoted the biosynthesis of flavonoids, and enhanced plant tolerance to salt stress [82]. Transgenic *Arabidopsis* overexpressing *AtNAC078* increased the content of flavonoids under strong light conditions by upregulating the expression of *CHS*, *F3′H*, *DFR*, and *LDO* [83]. *MdNAC52* promoted the biosynthesis of flavonoid compounds (anthocyanins and procyanidins) in apples by binding and activating the promoters of *MdMYB9*, *MdMYB11*, and *LAR* [84]. *Arabidopsis AtDOF4* upregulated the expression of structural genes of *DFR*, *LDOX*, *TT19*, and *PAP1* to increase the content of flavonoids in plants [85]. Apple callus overexpressing *MdWRKY11* was able to increase the expression of *F3H*, *FLS*, *DFR*, *ANS*, and *UFGT* and promote the biosynthesis of flavonoids and anthocyanins [86].

### 4.3. Non-Coding RNA Regulates Flavonoid Biosynthesis

Non-coding RNA, including lncRNA (long non-coding RNAs) and microRNA, played important roles in regulating flavonoid biosynthesis [87]. lncRNAs may act as precursors and endogenous target mimics of miRNAs to indirectly regulate protein-coding genes (PCgenes) [87]. Two lncRNAs, XR_001591906 and MSTRG.9304, were found to regulate the expression of the *CHS* gene in flavonoid biosynthesis during peanut seed development [88]. miRNAs directly cleave structural genes (SG) for flavonoid synthesis, thereby negatively regulating the accumulation of flavonoids, including miR396-targeting UFGT, miR172-targeting 4CL, and miR829.1-targeting CHS [89]. The miRNA-directed cleavage of TFs involved in flavonoid synthesis through miRNA–TF–SG regulatory networks such as miR156–SPL–F3H, miR828/TAS4–MYBs–DFR, and miR858–MYBs–CHS/FLS [89,90].

## 5. Pharmacological Properties of Flavonoids

### 5.1. Antioxidant Activity

**Figure 3:**
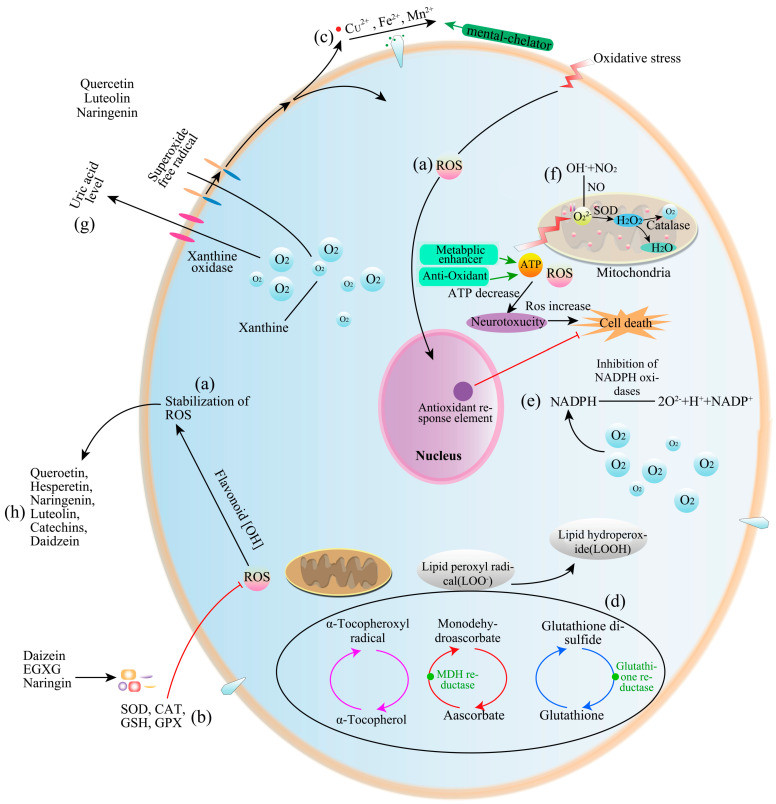
Mechanisms of flavonoid antioxidant activity in vitro. (**a**) Direct scavenging of reactive oxygen species (ROS); (**b**) Activation of antioxidant enzymes; (**c**) Activation of metal-chelating activity; (**d**) Increasing α-tocopheryl radical levels; (**e**) Inhibiting NAPDH oxidases; (**f**) Mitigation of oxidative stress caused by NO; (**g**) Increasing uric acid levels; (**h**) Increasing antioxidant properties of low-molecular-weight antioxidants.

The molecular mechanisms of antioxidant action contain the following processes (Figure 3). The antioxidant activity of flavonoids depends on their stable roles in different matrices, as well as the position and number of hydroxyl groups in their structures (Table A1) [91,92]. The mechanisms of antioxidant activity mainly comprise the following three processes: (1) Direct scavenging of ROS. In vitro, flavonoid antioxidant activity depends on the arrangement of hydroxyl groups. The B-ring, with its orthodihydroxy structure, allows flavonoid phenoxyl radicals to participate in electron delocalization, leading to the dislocation of an electron in the B-ring, which exhibits antioxidant activity. These structures enhance antioxidant activity [9]. Antioxidant activity was found to be increased in vitro through the polymerization of flavonoid monomers, such as proanthocyanidins (also called condensed tannins), and the polymers of catechins, which displayed excellent antioxidant activity due to the large number of hydroxyl groups in their molecules [93]. Moreover, free radical scavenging activity was found to be promoted by double bonds in the phenolic ring and hydroxyl side chains and through the glycosylation of anthocyanidins [9]. (2) The activation of antioxidant enzymes. Some flavonoids can suppress the activities of free radical-generating enzymes such as nitric-oxide synthase and xanthine oxidase [94]. Furthermore, flavonoids activate antioxidant enzymes, including the induction of phase II detoxifying enzymes (e.g., NAD(P)H-quinone oxidoreductase, glutathione S-transferase, and UDP-glucuronosyltransferase), which are the major defense enzymes against electrophilic toxicants and oxidative stress. The electrophile-responsive element (EpRE) is distributed in the 5′-regulatory region of a number of genes encoding phase II enzymes. Flavonoids have been found to activate the EpRE-mediated gene expression and display redox properties [95]. (3) The inhibition of oxidases. Flavonoids exert antioxidant activity by inhibiting the activities of xanthine oxidase (XO) and protein kinase C, which catalyze the production of superoxide anion. Zeng et al. reported that fisetin reduced oxidative damage by inhibiting XO activity [96]. Furthermore, flavonoids can inhibit the activities of cyclooxygenase, NADH oxidase, microsomal monooxygenase, and lipoxygenase [10,97]. (4) Metal-chelating activity. Hydroxyflavones may generate complexes with metal cations, and their chelating properties differ significantly depending on the number and position of the hydroxyl substituents [98]. Specific flavonoids can decrease the toxicity of redox-active transition metal ions [99]. Lesjak et al. revealed that quercetin, catechin, and rutin displayed high antioxidant activity against Fe (III) [100]. Kaempferol can chelate Cu(II) ions, suppress the formation of oxidants and radicals, and possess strong antioxidant properties [101]. Numerous flavonoids can generate stable metal complexes between the carbonyl moiety and their OH groups. Quercetin possesses three potential bidentate binding sites (α-hydroxy-carbonyl, β-hydroxy-carbonyl, and catechol), which can generate stable metallic complexes. In addition, catechin can chelate a large number of metal ions, including Mn (VI), Fe(II), Fe(III), Cu(II), Zn(II), and Al(III) [102]. (5) Increasing the level of α-tocopherol radicals. The oxidation of low-density lipoprotein (LDL) represents a free radical-driven lipid peroxidation process, which is associated with human health [103]. Flavonoids act as hydrogen donors to α-tocopherol radicals and have great potential to delay LDL oxidation through interaction with the α-tocopherol radicals. Torello et al. (2021) reported that several flavonoids (e.g., quercetin, epigallocatechin gallate, and naringin) showed strong inhibitory activity against LDL oxidation in vitro [104]. (6) Mitigating oxidative stress caused by nitric oxide. Nitric oxide (NO) plays an important role in maintaining the dilation of blood vessels, and a loss of NO causes oxidative stress in the vasculature [105]. The interaction of NO and O^2−^ forms the cytotoxic product peroxynitrite, which is a powerful oxidant capable of causing pathological damage. Flavonoids can inhibit the production of NO in several lipopolysaccharide-activated cells by inhibiting inducible NOS expression [106]. Among flavonoids, quercetin can suppress the production of NO and vascular endothelial growth factor (VEGF) [107].

**Table 1:** Pharmacological activities of flavonoids.

Flavonoids	Classification	Pharmacological Activity	Sources of Plant	References
Proanthocyanidins	anthocyanins	antioxidant, anti-inflammatory, antibacterial, antifungal and anti-cardiovascular	grapes, apples, sorghum, cherries, and other natural plant	[93]
Cyanidin	anthocyanins	anti-inflammatory, antiviral, and anticancer	black rice, black beans, purple potatoes, blueberries	[108]
Curcumin	curcuminoids	anti-inflammatory and anticancer	Curcuma longa	[109]
Methyl chalcone	chalcones	anti-inflammatory and anticancer	apple, citrus, soybean, ginger, mulberry	[110]
Trans-chalcone	chalcones	anti-inflammatory and anticancer	apple, citrus, soybean, ginger, mulberry	[110]
Xanthohumol	chalcones	anti-cardiovascular and antiviral	Humulus lupulus	[111]
Licochalcone	chalcones	antibacterial and antifungal	Glycyrrhiza uralensis	[112]
Catechin	flavanols	antioxidant, anti-inflammatory, antiviral, and anti-cardiovascular	Camellia sinensis	[101,102,113]
Epigallocatechin gallate	flavanols	antioxidant, antibacterial, antifungal, anti-cardiovascular, and antiviral	Camellia sinensis	[104,114,115]
Naringin	flavanones	antioxidant, anti-inflammatory, anti-cardiovascular, and antiviral	lemons, oranges, grapefruits, citrus	[104,110,116,117,118,119]
Hesperidin	flavanones	anti-inflammatory, anti-cardiovascular, and antiviral	lemons, limes, oranges, grapefruits, citrus	[116,117,120,121]
Diosmin	flavanones	anti-inflammatory	citrus fruits	[122]
Orientin	flavanones	anti-inflammatory	Trollius chinensis, Cajanus cajan, Crataegus laevigata	[123]
Vitexin	flavanones	antioxidant, anti-inflammatory, and anticancer	Ficus deltoid, Spirodela polyrhiza	[123]
Acacetin	flavanones	anti-cardiovascular, anticancer, and antiviral	Acacia farnesiana	[124,125]
Silymarin	flavanones	antioxidant, anti-cardiovascular, and antiviral	Silybum marianum	[126,127]
Liquiritigenin	flavanones	anti-inflammatory, antiviral, and anticancer	Glycyrrhiza uralensis	[128]
Isorhamnetin	flavanones	antiviral and anticancer	Ginkgo biloba, Hippophae rhamnoides	[125]
Apigenin	flavones	antibacterial, antifungal, and antiviral	Apium graveolens	[129,130,131,132]
Morin	flavones	antioxidant and anti-inflammatory	Cudrania cochinchinensis, Maclura pomifera	[133]
Baicalin	flavones	Anti-cardiovascular, antibacterial, and antifungal	Scutellaria baicalensis	[114,134]
Luteolin	flavones	anti-inflammatory, anti-cardiovascular, and antiviral	Dracocephalum integrifolium, Lonicera japonica, Capsicum annuum	[132,135]
Fisetin	flavonols	antioxidant	strawberry, apple, onion, cucumber, and other fruits and vegetables	[96]
Quercetin	flavonols	antioxidant, anti-inflammatory, anti-cardiovascular, antibacterial, and antifungal	vegetables, fruit, seeds, nuts, tea, and red wine	[100,102,107,120,136,137,138]
Rutin	flavonols	antioxidant, anti-inflammatory, and antiviral	rue, tobacco, jujube, apricot, orange, tomato, buckwheat, and citrus fruits	[101,120,126,127]
Kaempferol	flavonols	antioxidant, anti-inflammatory, antibacterial, antiviral, and anticancer	fruits, vegetables, herbs, and other natural plants	[101,133,139]
Myricetin	flavonols	antioxidant, anti-inflammatory, and anti-cardiovascular	Myrica rubra	[133,140,141]
Glabrol	isoflavane	antibacterial and antifungal	Glycyrrhiza uralensis	[112]
Genistein	isoflavone	antioxidant, antifungal, antiviral, and anticancer	soybeans and other plants	[120,142,143]

### 5.2. Anti-Inflammatory Action

Inflammation has many causes, including infections, injuries, and diseases [144]. Chronic inflammation is a common pathological basis for age-associated diseases such as cardiovascular disease, diabetes, cancer, and Alzheimer’s disease [145]. Among these flavonoids, apigenin can reduce the steady-state mRNA levels induced by TNF-α and can downregulate the expression of intercellular adhesion molecule-1 (ICAM-1), E-selectin, and vascular cell adhesion molecule-1 (VCAM-1) to endothelial cells. Yang et al. reported that many flavonoids reduced the expression of pro-inflammatory cytokines (e.g., TNF-α, IL-1β, IL-8, IL-6, and monocyte chemoattractant protein-1 (MCP-1)) in Jurkat T cells, peripheral blood mononuclear cells, and RAW macrophages. Quercetin and catechins were found to increase IL-10 production through the combined inhibition of TNF-α and IL-1β [113]. Quercetin suppressed the activity of heat shock factor (HSF) and reduced heat-induced damage [136]. Furthermore, several flavonols (e.g., morin, quercetin, kaempferol, and myricetin) could inhibit the activity of lipoxygenase [133]. Many flavonoids could decrease the production of arachidonic acid and suppress the activities of phospholipase A_2_, cyclooxygenase, and NOS, which further decreased the production of key inflammatory substances (prostaglandins, leukotrienes, and NO) [146]. In addition, flavonoids could inhibit the production of arachidonic acid metabolites and chemokines and decrease leukocyte infiltration and edema [147]. Moreover, flavonoids could chelate metal iron, suppress the activation of the complement system, and lower inflammation [148]. Genistein reduced airway hyper-responsiveness, ovalbumin-induced bronchoconstriction, and pulmonary eosinophilia in a guinea pig model of asthma and also suppressed the inflammatory response and joint destruction in collagen-induced arthritic mice. Quercetin, rutin, and hesperidin decreased chronic inflammation in an experimental model, and the results further showed that rutin played a key role in the chronic phase [120]. Diosmin and hesperidin could inhibit leukotriene B4 (LTB4) biosynthesis, which reduces colitis in a trinitrobenzenesulfonic acid (TNBS)-induced colitis rat model [122]. Naringenin displayed anti-inflammatory activity by inhibiting pro-inflammatory cytokines and reducing leukocyte infiltration. Several chalcones (e.g., trans-chalcone, hesperidin, and methyl chalcone) displayed inhibitory activity against pro-inflammatory cytokines [110]. Zhong et al. revealed that the flavonoids (vitexin, orientin, and rutin) from Tartary buckwheat sprout could reduce NO production by inhibiting *iNOS* and *COX2* expression in lipopolysaccharide (LPS)-induced RAW cells and in male BALB/c mice [123]. Quercetin and myricetin protected 661 W cells and the cone photoreceptor cell line from the toxic effects of H_2_O_2_. They also enhanced the gene expression of M and S opsins under oxidative stress [140].

### 5.3. Cardiovascular Action

Flavonoids used as cardioprotective agents can regulate oxidative stress and inflammation, commonly cause vasodilation, and regulate endothelial cell apoptosis [149]. Recent studies have revealed that dietary flavonoid intake can effectively reduce the risk of cardiovascular death in adult Americans [150]. In vitro and in animal models, some flavonoids have vasodilatory effects, improve endothelial dysfunction and insulin resistance, exert antiplatelet aggregation and atherosclerotic protective effects, and lower blood pressure [151]. Flavonoids were found to prevent hepatic steatosis, dyslipidemia, and insulin sensitivity, primarily by inhibiting hepatic fatty acid synthesis and increasing fatty acid oxidation [152]. Several studies have demonstrated that naringenin, quercetin, and hesperetin possess vasodilator properties, and their results further showed that naringenin could reduce high blood pressure by promoting vasodilation [116,117]. Isoflavones seem to protect against inflammatory vascular diseases by inhibiting monocyte-endothelial cell adhesion [153]. Quercetin exerts cardioprotective effects of quercetin against ischemia-reperfusion injury in the heart and also exhibits atheroprotective activity [137]. Baicalin can improve cardiac dysfunction and inhibit apoptosis in the heart. Chrysin has been reported to possess antiplatelet activity [134]. Anthocyanins reduce the risk of myocardial infarction (MI) in humans, improve systolic blood pressure, and reduce the content of triglycerides, total cholesterol, and low-density lipoprotein cholesterol [154,155]. Several studies have demonstrated that acacetin plays an important role in regulating human arrhythmia [124]. Sun et al. (NIDAN) found that xanthohumol could increase PTEN expression and inhibit AKT/mTOR phosphorylation in isoprenaline-treated mice and exert a protective effect against ISO-induced myocardial hypertrophy and fibrosis [111]. Flavonoids extracted from *Myrica rubra* exerted cardioprotective effects by modulating the PI3K/Akt/GSK3β pathway to attenuate oxidative damage and cardiomyocyte apoptosis [141]. The sirtuin 1 (SIRT1) enzyme plays a key role in the regulation of many physiological functions, and its reduced expression often causes aging-related diseases such as myocardial hypertrophy, myocardial infarction, and endothelial dysfunction. A study by Testai et al. found that the long-term administration of the citrus flavonoid naringenin (NAR) (100 mg/kg/day) in mice resulted in enhanced SIRT1 expression, significantly reduced ROS production in myocardial tissue and significantly lowered levels of the cardiovascular inflammatory markers TNF-α and IL6, revealing that nutritional therapy with NAR may help improve myocardial aging and protect cardiac function [118]. Yang et al. also revealed that *Oxytropis falcata* flavonoid extracts protect against myocardial ischemia-reperfusion injury by downregulating the ROS-mediated JNK/p38MAPK/NFκB pathway to regulate inflammatory responses, oxidative stress, and apoptosis [156].

### 5.4. Antibacterial and Antifungal Action

Flavonoids exert their antibacterial activity through several mechanisms, including bacterial membrane disruption, the inhibition of biofilm formation, the suppression of nucleic acid and ATP biosynthesis, and the disruption of electron transport (Table A1) [6,157]. Quercetin, apigenin, naringenin, chrysin, genistein, kaempferol, daidzin, and daidzein were found to block the biofilm formation, and the results further showed that quercetin, myricetin, baicalein, and luteolin suppressed the DNA replication in bacteria [8,158]. The epigallocatechin gallate and baicalein suppressed the biosynthesis of ATP in bacteria [114]. Wu et al. [112] found that the flavonoids (e.g., glabrol, licochalcone A, licochalcone C, and licochalcone E) from licorice showed high efficiency against MRSA and displayed low cytotoxicity to mammalian cells. Among these flavonoids, glabrol exerts a bactericidal effect by increasing the permeability of the cell membrane and collapsing the proton motive force. Zhang et al. [159] also found that flavonoid extracts from *Coriolus versicolors* showed strong antibacterial activity against *Escherichia coli*, *Staphylococcus aureus*, and *Bacillus subtilis* in an experimental study. Yuan et al. [160] revealed that the cell membrane is the main site of flavonoids acting on Gram-positive bacteria, which likely involves the damage to phospholipid bilayers, the inhibition of the respiratory chain, or the ATP synthesis.

Various flavonoids have been extracted and studied to determine their antifungal activity and are expected to be promising, efficient, economical, and harmless drugs for inhibiting fungal infections in humans [161]. Several mechanisms have been proposed to explain the antifungal effects of flavonoids, including the plasma membrane disruption [162], induction of mitochondrial dysfunction [163], inhibition of cell wall generation [164], the suppression of cell division, the inhibition of RNA/protein biosynthesis, and the inhibition of efflux-mediated pumping systems [165]. Baicalein and apigenin were used as antifungal agents as they regulate ROS species, decrease lipid peroxidation, and block membrane disruption [50,130]. Glabridin inhibited the biosynthesis of the main components of fungi cell walls (β-glucans and chitin) [114]. Quercetin can inhibit oxidative phosphorylation and suppress the production of ROS [138]. Apigenin can control the cell cycle of fungi, while myricetin, quercetin, kaempferol, naringenin, genistein, and luteolin suppress the biosynthesis of DNA, RNA, and protein [166]. Li et al. [167] revealed that some flavonoids (glcyrrhiza, glabra, isoflavones, and chalcone) induced significant bactericidal effects by regulating the expression level of phosphatidylserine decarboxylase, which caused a loss of mitochondrial membrane potential and cell membrane disruption. Furthermore, baicalein and wogonin extracted from Scutellaria roots could induce apoptosis-like programmed cell death through the overproduction of ROS. Baicalein displayed a strong antifungal activity against *Trichophyton rubrum*, *Trichophyton mentagrophytes*, *Aspergillus fumigatus*, and *Candida albicans*, and wogonin inhibited all fungi except *Candida albicans*.

### 5.5. Antiviral Action

Flavonoids are known to be effective antivirals as they block virus attachment, preventing viruses from entering host cells and interfering with the replication, transcription, and translation of virus genomic DNA [168]. Roschek et al. [169] revealed that flavonoids can attach themselves to the surface proteins of viruses, inhibit viruses from entering the host cells, modulate the immune system, and reduce viral load. In vitro and in vivo studies have revealed that apigenin exhibits a wide range of antiviral effects against RNA and DNA viruses, such as herpes simplex virus type 1 and type 2, African swine fever virus, hepatitis B virus, and hepatitis C virus [131]. For example, baicalein can block the replication of the avian influenza H5N1 virus in humans [170]. Luteolin can inhibit HIV-1 reactivation, and genistein suppresses HIV-1 infection in CD4 + T cells and macrophages [135]. Kaempferol can block the HIV-1 replication in host cells and prevent herpes simplex virus types 1 and 2 from entering them [139]. Badshah et al. [171] comprehensively reviewed many flavonoids that showed antiviral activities in different testing environments, such as in vitro, in vivo (mouse model), and in silico.

The anti-inflammatory activity of flavonoids also influences cancer, carcinogen inactivation, anti-proliferation, cell cycle arrest, the induction of apoptosis, and the inhibition of angiogenesis [172]. Epidemiological studies revealed that dietary flavonoid intake could decrease the risk of breast, lung, colon, prostate, and pancreas tumors [173]. Several flavonoid compounds, including quercetin, rutin, hesperetin, silymarin, xanthohumol, 7,7′-dimethoxyagastisflavone, chrysoplenetin and chrysophanol D, formononetin, genistein, cyaniding and peonidin isolated from many medicinal plants, exert genoprotective, cytotoxic, anti-proliferative and/or proapoptotic actions in different tumoural cell lines [126,127]. Hirchaud et al. revealed that liquiritigenin isolated from licorice promoted apoptosis in HeLa cells by increasing the p53 and Bax gene expression, decreasing Bcl-2 gene expression, releasing cytochrome c, and elevating the activity of caspase-9 and -3 [128]. Flavonoids inhibit angiogenesis and metastasis by regulating the expression levels of *VEGF* and *TGF-b1* genes [131]. In addition, flavonoids isolated from Dimorphandra mollis and Croton betulaster inhibited the proliferation of human glioblastoma cells and reduced the expression levels of *VEGF* and *TGF-b1* genes [174]. Lewis et al. found that the therapy combining SAHA and EGCG not only significantly reduced the expression levels of miR-221/222 but also increased p27 and ER in carcinogenic α cells and the expression level of tumor suppressor genes [175]. Moreover, flavonoids have the ability to regulate non-coding microRNAs (miRNAs). The ability to alter miRNA levels through different mechanisms, either by inhibiting oncogenic miRNAs or activating tumor-suppressive miRNAs or by affecting transcription and epigenetic miRNA processing in TNBC [176].

There are two flavonoid compounds, isorhamnetin, and acacetin, that suppress the proliferation of human breast cancer cells [125]. The isoflavone genistein promotes breast cancer cell arrest at the G2/M phase and subsequent ROS-dependent apoptosis. Kaempferol has been shown to induce apoptosis via cell cycle arrest in human breast cancer MDA-MB-453 cells [142]. Genistein acts as a potential anticancer agent against multiple cancers such as breast cancer, prostate cancer, and ovarian cancer by inducing cell cycle G2/M phase arrest [143]. Naringenin can inhibit ROS formation, improve antioxidant enzyme activity such as SOD, CAT, and GSH, and further inhibit the proliferation and migration of MG-63 human osteosarcoma cells [119]. Hesperidin suppresses MG-63 cell cycle progression and activates the apoptosis of cancer cells [121]. Epigallocatechin-3-gallate (ECGG) causes growth arrest and cell death in prostate cancer cells [177]. In in vitro research, quercetin caused cell_cycle arrest, DNA damage, and cell growth inhibition in several cancer cell lines in vitro research, such as leukemia cancer, colon cancer, breast cancer, and ovarian cancer [115]. Apigenin and luteolin cause alterations to ROS signaling and induce apoptosis in several ovarian cancer cell lines, such as A2780, OVCAR-3, and SKOV-3 [132]. Cyanidin blocks cancer cell proliferation and induces apoptosis in the human epithelial colorectal adenocarcinoma cell line (Caco-2) [108]. In one study, resveratrol combined with grape seed proanthocyanidins significantly reduced the DNMT and HDAC activities of breast cancer cell lines [178]. In addition, resveratrol and procyanidins can synergistically inhibit the growth of breast cancer cells [179]. Flavonoids and other polyphenols act as epigenetic modifiers in breast cancer. Moreover, Mirza et al. reported that EGCG, genistein, curcumin, resveratrol, lutein A and gingival sterone exhibit cancer prevention effects through the epigenetic regulation of tumor suppressor genes [109].

## 6. Applications of Flavonoids in Cosmetics and Foods

### 6.1. Applications of Flavonoids in Cosmetics

Oxidative stress can cause changes in skin pigmentation changes and facial aging [180]. Thus, antioxidant supplements are considered the best approach to both the prevention and treatment of the above-mentioned issues [181]. Flavonoids are widely used in common cosmetics primarily due to their antioxidant and soothing properties [182]. The cosmetic applications of flavonoids comprise three aspects: sun protection, antiaging effects, and anti-inflammatory effects [183]. (1) Sun protection. The chromophores of flavonoids can increase light absorption, absorb light in the UV/blue spectral region, and reduce oxidative stress damage from sunlight [184]. Silymarin, a mixture of flavonolignans, has antioxidant, anti-inflammatory, and immunomodulatory properties and has led to the prevention of photocarcinogenesis in mice. Linarin, a flavone glycoside from *Buddleja cardioids*, was tested on guinea pigs and shown to have a sun protection factor (SPF) value of 9, thus providing remarkable protection from UV damage [185]. The quercetin and rutin from *Moringa oleifera* exhibited an SPF value of 2 [186]. The O/W formulation with anthocyanin from raspberry and blackberry obtained a high SPF value of 15.8 [187]. Anthocyanins activated transcription factor Nrf2 and induced the production of various antioxidant enzymes [188]. Finally, acacetin can prevent UVB-induced MMP-1 expression, which leads to skin photoaging, and may therefore have therapeutic potential as an anti-wrinkle agent to improve skin health [189]. (2) Antiaging effects. Flavonoids play important roles in protecting against the signs of aging, especially in the skin [190]. Micek et al. [191] reported that 3% taxifolin cream improved the viscoelasticity of aging skin with a better penetration rate. Water/ethanolic extracts from various parts of *Nymphaea lotus* are rich in flavonoids, which are usually used for homemade self-care products [189]. Water lily extracts contain excess flavonoids, which inhibit matrix metalloproteinase-1 and cause skin aging via extracellular matrix decompositions [192]. (3) Anti-inflammatory effects. Flavonoids inhibit phospholipase and cyclooxygenase activities and regulate immune cell migration and cytokines production [193]. A previous study revealed that the polymethoxyflavones from the *Kaempferia parviflora* inhibited pro-inflammatory mediators induced by TNF-α, such as cyclooxygenase-2 (COX-2), IL-6, interleukin (IL)-1β, mitogen-activated protein kinase (MAPK), nuclear factor-kappa B (NF-κB), and activator protein 1 (AP-1) [194,195]. Galangin, a specific flavonoid compound distributed in *Alpinia galanga*, has protective properties against human skin fibroblasts, which also exhibit an anti-inflammation effect by inhibiting NF-κB and increasing heme oxygenase-1 [189,196].

### 6.2. Application of Flavonoids in Foods

Food additives, derived from either plants or animals or produced via artificial synthesis, are added to foods and related products to enhance their flavor, taste, and freshness [197]. However, the excessive consumption of synthetic additives may cause potential risks to human health. Flavonoids, as the most important group of phenolics, are widely distributed in many vegetables and fruits and are used in food processing to decrease the use of synthetic chemicals and improve human health [198]. The content of flavonoids varies between plants and between organs of the same plant. Several horticultural crops, cabbage, carrots, spinach, mushrooms, peaches, strawberries, orange juice, and white wine, contain relatively low flavonoid content (less than 10 mg/kg) [198]. The content of flavonoids is lower than 50 mg/kg in lettuce, beans, red pepper, tomato, grapes, and tea and is higher than 50 mg/kg in broccoli, kale, French beans, celery, and cranberries [199]. The highest content is found in fruits such as berries (33.63 mg/100 g anthocyanins in strawberries and 13.52 mg/100 g anthocyanins in blueberries), apples (184 mg/200 g quercetin and 180 mg/200 g epicatechin), and citrus fruit (292 mg/500 mL hesperidin in orange juice) [198]. Moreover, flavonoids are used to extend the shelf life of many foods and preserve many foods due to their antimicrobial and antioxidant properties [200]. With the development of the economy and the improvements in people’s living standards, natural compounds have shown greater activity than synthetic chemicals because the body can accept natural compounds [201]. Flavonoids are used as food preservatives to prevent fat and oil oxidation, feed animals as supplements, protect various enzymes and vitamins, and inhibit microbial growth in foods [198,202]. A previous study revealed that flavonoids could inhibit lipid oxidation in red meats and poultry and retard spoilage due to microorganism growth in meats thanks to their antioxidant and antimicrobial properties [200,203].

## 7. Omics Research and Flavonoid Nanoparticles

Omics research attempts to comprehensively understand the biological molecules in an organism at a particular functional level, such as the genome, transcriptome, or proteome [204]. Complete genomic sequences and annotations provided important information, including promoter sequences, gene sequences, gene structure, and predicted gene functions. Recently, a combination of transcriptomic and metabolomic analyses has represented an effective approach to exploring the functions of genes associated with metabolism [205]. Yuan et al. showed that metabolome analysis detected 124 different flavonoid metabolites, and 30 different genes involved in flavonoid biosynthesis were identified through transcriptome analysis [206]. Integrated transcriptomic and metabolomic data revealed that anthocyanin biosynthetic genes exhibited the differential expression patterns between purple- and green-skinned fruit of *Ficus carica* [207]. Similarly, the accumulation of malvidin 3-*O*-glucoside and delphinidin 3-*O*-glucoside was associated with the reddening of the jujube peel, which may be correlated with an increase in the expression levels of three *UFGT* genes [208]. Combined fruit transcriptomic and metabolomic analyses of fruit have uncovered six candidate genes (*AaF3H*, *AaLDOX*, *AaUFGT*, *AaMYB*, *AabHLH*, and *AaHB2*) and seven flavonoid compounds that are closely associated with the pigmentation of red- and green-fleshed cultivars of *Actinidia arguta* [209]. Thus, the combination of transcriptomic (proteomics) and metabolomic analyses could be used to investigate the spatial distributions and dynamics of flavonoid metabolites and their corresponding spatiotemporal gene expression.

Although flavonoids are thought to have beneficial effects on human health, such as antioxidant, anti-inflammatory, and anticancer properties, the use of flavonoids in disease treatment is not satisfactory due to low solubility, poor absorption, and rapid metabolism [210]. Nanotechnology can solve the issue with flavonoids, namely, low water solubility, which plays a major role in low bioavailability. For example, fisetin, as a hydrophobic dietary flavone found in strawberries, apples, cucumbers, and onions, possessed low aqueous solubility (less than 1 mg/mL) and resulted in low bioavailability, and has limited its use [211]. Moreover, different types of flavonoid nanoparticles have different therapeutic effects on different diseases. Dobrzynska et al. summarized the effects of the different flavonoid nanoparticles on cancer therapy by enhancing their anti-tumor effects or reducing the systemic toxicity of drugs [212]. Siddiqui et al. showed that the polylactic acid-polyethylene glycol (PLA-PEG) with encapsulated EGCG nanoparticles were more than 10 times more effective against 22Rr1 prostate cancer cells than free EGCG [213]. Quercetin-loaded PEG nanoparticles were found to prolong the circulation time of quercetin in the bloodstream and increase its solubility and stability. Tan et al. revealed that the PEG-derivatized phosphatidylethanolamine nanomicelles increased the anticancer activity of quercetin and were more effective against A549 lung cancer cells than free-quercetin [214]. Genistein-loaded TPGS-b-PCL (d-α-tocopheryl polyethylene glycol 1000 succinate-poly(ε-caprolactone)) was found to inhibit HeLa cervical tumor cells growth and possessed a higher level of cytotoxicity in comparison with genistein-loaded PCL nanoparticles [212]. In addition, apigenin nanoparticles, including apigenin loaded in PLGA nanoparticles and apigenin encapsulated in PLGA nanoparticles, showed an anti-proliferative effect against A475 skin cancer cells and delayed the development of hepatocellular carcinoma in rats [212]. Nanonargenin works by inhibiting both the PI3K and MAPK paths and by restricting ER alpha to the cytoplasm to lessen the proliferation of Tam-RMC cells [215].

## 8. Conclusions

Flavonoids represent the main class of plant secondary metabolites and occur in the different tissues and organs of various plant species. The elucidation of their biosynthetic pathways, as well as their regulation by transcription factors and non-coding RNA, has enabled researchers to employ metabolic engineering to synthesize diverse flavonoids with valuable applications. Flavonoid compounds are classified into seven subgroups due to modifications to their basic skeletons, including flavones, flavanones, isoflavones, flavonols or catechins, and anthocyanins. Flavonoids, as potential candidates for bioactive compounds, are widely used in food processing and the cosmetics and pharmaceutical industries to improve human health due to their antioxidant and free radical scavenging properties. Moreover, the biological activities of flavonoid compounds, including their antioxidant effects, antimicrobial effects, anticancer effects, cardioprotective effects, anti-inflammatory effects, and skin protective effects, were addressed in this review. In addition, we summarized the mining of new functional genes metabolites through omics research, and the engineering of flavonoids using nanotechnology was also summarized in this review. This review provides a reference for basic and applied research on flavonoid compounds.

## 9. Future Perspectives

### 9.1. Mining of Functional Genes

Plant genomes and transcriptomes were widely used to investigate gene information, including gene locations, gene structure, and gene expression patterns. Integrated transcriptome (proteome) and metabolome analyses have revealed the dynamic changes in flavonoid compounds and the corresponding functional genes and transcription factors. The yeast one-hybrid and yeast two-hybrid systems are considered valuable and straightforward techniques for studying the interactions between transcription factors and the promotion of functional genes and transcription factors. DNA molecular markers and a genome-wide association study (GWAS) were used to identify the relationships between genotypes and phenotypes and explore the variation in genomic loci associated with the important agronomic traits and detected key genes.

### 9.2. Extraction and Utilization of Bioactive Ingredients

Sample extraction techniques severely block the isolation and extraction of individual flavonoid compounds in plants, which severely restricts the development of medicines. In addition, the pharmacological mechanisms of flavonoids and their derivatives in many plants are still unclear due to the lack of animal studies and clinical trials. With the innovations of new extraction techniques and the development of molecular biology, studies on flavonoids and their derivatives mainly focus on their isolation and extraction, metabolic pathway analysis, molecular regulatory mechanisms, and potential applications in pharmacy and health services research using nanotechnology.

## Data Availability

Not applicable.

## Appendix A

**Table A1:** The Biological Activity and Mechanism of Action of Flavonoids.

Flavonoids	Biological Activity	Mechanism of Action	References
Proanthocyanidins	antioxidant activity	polymerizing flavonoid monomer and Catechin polymer	[93]
Fisetin	antioxidant activity	inhibiting the activities of oxidase	[10,96,97]
Hydroxyflavones	antioxidant activity	generating complexes with metal cations	[98]
Kaempferol	antioxidant activity	chelate Cu(II) ions	[101]
Catechol	antioxidant activity	generating stable metallic complexes	[102]
Quercetin	antioxidant activity	interacting with α-tocopherol radicals	[104,106,107]
Epigallocatechin Gallate Naringin	antioxidant activity	interacting with α-tocopherol radicals	[104]
Apigenin	antiinflammatory action	reducing the steady-state mRNA levels induced by TNF-α	[145]
Quercetin catechins	antiinflammatory action	reducing the expression of pro-inflammatory cytokines	[113,133]
Kaempferol Myricetin	antiinflammatory action	inhibit the activity of lipoxygenase	[133]
Diosmin Hesperidin	antiinflammatory action	inhibiting leukotriene B4 biosynthesis	[122]
Chalcones	antiinflammatory action	inhibiting proinflammatory cytokines activity	[110]
Vitexin Orientin Rutin	antiinflammatory action	inhibiting iNOS and COX2 expression	[123]
Naringenin Hesperetin	anticardiovascular action	reducing high blood pressure by promoting vasodilation	[116,117]
Quercetin	anticardiovascular action	protecting the heart against ischemia-reperfusion injury via the cardioprotective effects of quercetin.	[116,117,137]
Isoflavones	anticardiovascular action	inhibiting monocyte-endothelial cell adhesion	[153]
Anthocyanins	anticardiovascular action	improving systolic blood pressure, and reduce the content of triglycerides, total cholesterol, and low-density lipoprotein cholesterol	[154,155]
Xanthohumol	anticardiovascular action	increasing PTEN expression and inhibited AKT/mTOR phosphorylation	[111]
Flavonoid	anticardiovascular action	modulating the PI3K/Akt/GSK3β pathway or downregulating the ROS-mediated JNK/p38MAPK/NFκB pathway	[141,156]
the citrus flavonoid naringenin	anticardiovascular action	enhancing SIRT1 expression, reduced ROS production	[118]
Quercetin	antibacterial action	blocking the biofilm formation and suppressed the DNA replication	[8,138,158,166]
Apigenin Naringenin Chrysin Genistein Kaempferol Daidzin	Antibacterial action	blocking the biofilm formation or regulate ROS species, decrease lipid peroxidation	[8,129,130,158,166]
Baicalein Luteolin Myricetin	antibacterial action	suppressing the DNA replication or suppressed the biosynthesis of ATP	[8,129,130,158]
Epigallocatechin gallate	antibacterial action	suppressing the biosynthesis of ATP	[114]
Glabrol	antibacterial action	increasing the permeability of the cell membrane and collapsing the proton motive force	[112]
Glabridin	antifungal action	inhibiting the biosynthesis of the main components of fungi cell walls	[114]
Myricetin Kaempferol Naringenin Genistein Luteolin	antifungal action	suppressing the biosynthesis of DNA, RNA, and protein	[166]
Glcyrrhiza Glabra Isoflavones Chalcone	antifungal action	regulating the expression level of phosphatidylserine decarboxylase	[167]
Apigenin	antiviral action	inhibiting viruses from entering the host cells, modulate the immune system, and reduce viral load	[131,169]
Baicalein	antiviral action	blocking the replication of the avian influenza H5N1 virus	[170]
Luteolin	antiviral action	inhibiting HIV-1 reactivation	[135]
Kaempferol	antiviral action	blocking the HIV-1 replication	[139]
Liquiritigenin	promoted apoptosis	increasing the *p53* and *Bax* gene expression, decreasing *Bcl-2* gene expression	[128]
Isoflflavone genistein	suppress the proliferation	promoting breast cancer cell arrest at the G2/M phase and subsequent ROS-dependent apoptosis	[125,143]
Kaempferol	suppress the proliferation	inducing apoptosis via cell cycle arrest MDA-MB-453 cells	[125,142]
Naringenin	inhibit the proliferation and migration	inhibiting ROS formation	[119]
Apigenin Luteolin	induce apoptosis	causing the alterations to ROS signaling	[132]
